# Desigualdades na tendência da sífilis congênita no município de Niterói, Brasil, 2007 a 2016

**DOI:** 10.26633/RPSP.2020.8

**Published:** 2020-02-04

**Authors:** Andressa Lohan dos Santos Heringer, Helia Kawa, Sandra Costa Fonseca, Sandra Mara Silva Brignol, Loren Angelica Zarpellon, Ana Cristina Reis

**Affiliations:** 1 Universidade Federal Fluminense (UFF), Instituto de Saúde Coletiva Departamento de Epidemiología e Bioestatística Niterói (RJ) Brasil Universidade Federal Fluminense (UFF), Instituto de Saúde Coletiva, Departamento de Epidemiología e Bioestatística, Niterói (RJ), Brasil.; 2 Escola Politécnica de Saúde Joaquim Venâncio (EPSJV/FIOCRUZ) Laboratório de Educação Profissional em Informações e Registros em Saúde Rio de Janeiro (RJ) Brasil Escola Politécnica de Saúde Joaquim Venâncio (EPSJV/FIOCRUZ), Laboratório de Educação Profissional em Informações e Registros em Saúde, Rio de Janeiro (RJ), Brasil.

**Keywords:** Sífilis congênita, iniquidade social, estudos de séries temporais, vigilância, Brasil, Syphilis, congenital, social inequity, time series studies, surveillance, Brazil, Sífilis congénita, inequidad social, estudios de series temporales, vigilancia, Brasil

## Abstract

**Objetivo.:**

Descrever a distribuição temporal e as características epidemiológicas da sífilis congênita (SC) em Niterói, Sudeste do Brasil, de 2007 a 2016.

**Métodos.:**

Este estudo descritivo de série temporal da incidência de SC utilizou os dados do Sistema de Informação de Agravos de Notificação (SINAN) e do Sistema de Informações sobre Nascidos Vivos (SINASC). A amostra incluiu todos os casos notificados. Além disso, foi realizado um relacionamento probabilístico entre SINAN e SINASC para recuperar informações ignoradas. A série temporal foi estimada por regressão logarítmica, de acordo com variáveis sociodemográficas e de pré-natal.

**Resultados.:**

Identificaram-se 754 casos de SC no período estudado (incidência média de 11,9 casos/1 000 nascidos vivos). A incidência foi mais elevada em jovens (10 a 19 anos; 20 a 24 anos), participantes de cor preta e naquelas com baixa escolaridade e sem pré-natal. Do total de mulheres, apenas 57,6% obtiveram o diagnóstico de sífilis durante o pré-natal. O tratamento foi inadequado em 87,7% das mulheres. Apenas 12,2% dos parceiros foram tratados. Houve tendência crescente do agravo (16%/ano), que atingiu 23,2 casos/1 000 nascidos vivos em 2016. O crescimento foi mais acentuado em adolescentes do sexo feminino (25,2%/ano), raça/cor parda (16,8%/ano), indivíduos com baixa escolaridade (57,1%/ano) e mulheres que realizaram pré-natal (17,3%/ano); e, no período de 2012 a 2016, em mulheres com informação ignorada para a cor da pele.

**Conclusões.:**

As iniquidades sociais se destacaram na ocorrência de SC, com incidência crescente em jovens. É necessária a capacitação dos profissionais de saúde para o manejo da sífilis gestacional e uma atuação efetiva das políticas públicas sobre os determinantes sociais da sífilis.

## Abstract (en)

**Objective.:**

To describe the temporal distribution and epidemiologic characteristics of congenital syphilis (CS) cases in the city of Niterói, southeastern Brazil, from 2007 to 2016.

**Method.:**

This descriptive time series analysis of the incidence of CS used data from the Notifiable Diseases Information System (SINAN) and the Live Birth Information System (SINASC). The sample included all notified cases. A probabilistic matching was performed between SINAN and SINASC data to recover ignored information. The time series was estimated using logarithmic regression according to sociodemographic and prenatal care variables.

**Results.:**

There were 754 identified cases of CS in the study period (mean incidence: 11.9 cases/1 000 live births). The incidence was higher in younger women (10 to 19; 20 to 24 years) and in those with black skin, low schooling, and without prenatal care. Of the overall group, only 57.6% received a diagnosis of syphilis during prenatal care. Treatment was not adequate in 87,7%, and only 12.2% of partners were treated. SC incidence presented a growing trend of 16%/year, reaching 23.2 cases/1 000 living births in 2016. This growth was especially marked in female adolescents (25.2%/year), brown race/skin color (16.8%/year), women with low schooling (57.1%/year) and women who received prenatal care (17.3%/year); and, from 2012 to 2016, in women without information on skin color.

**Conclusions.:**

Social inequalities were linked to CS in the present sample. Also, increasing CS incidence was detected in youth. Health care professionals must be trained to manage gestational syphilis, and public policies must effectively address the social determinants of this condition.

## RESUMEN

**Objetivo.:**

Describir la distribución temporal y las características epidemiológicas de la sífilis congénita en Niterói, sureste de Brasil, de 2007 a 2016.

**Métodos.:**

Estudio descriptivo de series temporales sobre la incidencia de la sífilis congénita; se utilizaron datos del Sistema de Información de Enfermedades Notificables (SINAN) y del Sistema de Información de Nacimientos Vivos (SINASC). La muestra incluyó todos los casos reportados. Se realizó además una relación probabilística entre el SINAN y el SINASC para recuperar información ignorada. La serie temporal se estimó mediante regresión logarítmica, según variables sociodemográficas y prenatales.

**Resultados.:**

En el período estudiado se identificaron 754 casos de sífilis congénita (incidencia media de 11,9 casos/1 000 nacidos vivos). La incidencia fue mayor en las mujeres jóvenes (10-19, 20-24 años), mujeres de raza negra, con baja escolaridad y sin atención prenatal. Del total de mujeres, sólo se realizó el diagnóstico de sífilis durante la atención prenatal en 57,6%. El tratamiento fue inadecuado en 87,7% de las mujeres, solo el 12,2% de las parejas fueron tratadas. La enfermedad presentó una tendencia creciente (16%/año), y alcanzó 23,2 casos/1 000 nacidos vivos en 2016. El aumento fue más pronunciado en las adolescentes (25,2%/año), raza/color de piel morena (16.8%/año), mujeres con baja educación (57,1%/año), mujeres que recibieron atención prenatal (17,3%/año) y, de 2012 a 2016, en mujeres sin información sobre el color de la piel.

**Conclusiones.:**

En esta muestra se destacaron las desigualdades sociales en la aparición de sífilis congénita, con una incidencia creciente en las jóvenes. Se requiere capacitación de los profesionales de la salud en el manejo de la sífilis gestacional y la acción efectiva de políticas públicas sobre los determinantes sociales de la sífilis.

A sífilis congênita (SC) é uma doença que pode ser evitada pelo diagnóstico correto e pelo subsequente tratamento da gestante infectada (1). No entanto, dificuldades no diagnóstico oportuno e no tratamento adequado de gestantes e de seus parceiros (2, 3) têm levado a um aumento da sífilis em gestantes, com consequente aumento da transmissão vertical (4).

Nesse contexto, a Organização Pan-Americana da Saúde (OPAS) propôs renovar e articular as estratégias de enfrentamento da SC, definindo como meta para o ano de 2020 uma incidência de 0,5 casos/1 000 nascidos vivos (5). Para alcançar essa meta, é preciso implementar ações interprogramáticas que aumentem a cobertura da testagem de gestantes e mulheres em idade fértil e o tratamento imediato dos casos positivos (5, 6).

Mundialmente, a incidência de SC diminuiu de 5,4 casos por 1 000 nascidos vivos, em 2012, para 4,7 em 2016, exceto na Região das Américas e no Leste do Mediterrâneo (7). Em 2016, as maiores taxas se encontravam no continente africano (12/1 000 nascidos vivos) e as menores, na Europa (0,2/1 000 nascidos vivos) (7). Nas Américas, houve aumento de 3,07 para 3,19/1 000 nascidos vivos de 2012 a 2016 (7), embora alguns países tenham conseguido o certificado de eliminação da transmissão vertical de SC (5).

Na América Latina, o Brasil é responsável por 85% dos casos de SC (5), com aumento consistente ao longo dos anos (8): em 2007, a taxa era de 1,9 caso/1 000 nascidos vivos; em 2017, chegou a 8,6 casos/1 000 nascidos vivos, com 24 666 casos notificados (8). Naquele ano, a região Sudeste do país apresentou a maior taxa de incidência. Foi destaque o estado do Rio de Janeiro, com 18,8 casos/1 000 nascidos vivos (8) e taxas ainda mais altas em suas duas regiões metropolitanas: 22,1/1 000 nascidos vivos na Metropolitana I, que engloba a capital do estado e os municípios próximos, situados na baixada fluminense; e 26,9/1 000 nascidos vivos na Metropolitana II, também formada por municípios próximos da capital, porém do outro lado da Baía de Guanabara. Juntas, as regiões Metropolitanas I e II comportam três quartos da população do estado (9).

Os dados atuais sobre a SC no Brasil são provenientes do Sistema de Informação de Agravos de Notificação (SINAN), que apresenta subnotificação e elevado percentual de informações ignoradas ou em branco (4, 10-12). Isso limita o uso desse sistema em estudos sobre incidência e fatores relacionados ao aumento da SC. Entretanto, esse déficit pode ser compensado pelo relacionamento com outros sistemas de informação em saúde, como o Sistema de Informações sobre Nascidos Vivos (SINASC), o Sistema de Informações sobre Mortalidade (SIM) e o Sistema de Informações Hospitalares (SIH), aumentando a completude e a confiabilidade dos dados (10, 13). Por sua vez, a análise de séries temporais (12, 13, 13) pode revelar não só a evolução da incidência, mas também fatores associados às mudanças nos padrões de distribuição do agravo (15), sendo de grande utilidade para agravos de difícil controle, como a SC.

Diante disso, o objetivo deste estudo foi descrever a distribuição temporal e as características epidemiológicas da SC em residentes do município de Niterói, no Sudeste do Brasil, de 2007 a 2016.

## MATERIAIS E MÉTODOS

O presente estudo descritivo, de série temporal, utilizou dados secundários referentes ao município de Niterói, localizado na Região Metropolitana II do estado do Rio de Janeiro, Brasil. O período de análise foi de janeiro de 2007 a dezembro de 2016.

A área territorial do município de Niterói é de 133 919 km^2^. A população, estimada em 500 000 residentes, é totalmente urbana (16). O município tem índice de desenvolvimento humano (IDH) alto (0,837) e baixa vulnerabilidade social (16). A cobertura populacional da atenção básica era de 67% em 2007 e de 78% em 2016 (17).

A população do estudo contemplou todos os casos de SC notificados em residentes no município, incluindo abortos, óbitos fetais e nascidos vivos. Os dados foram obtidos do SINAN, com identificação dos casos de sífilis congênita a partir das fichas de investigação. Essas fichas contêm informações sociodemográficas, epidemiológicas, laboratoriais e clínicas preenchidas por profissionais de saúde. Também foi utilizado o banco de dados do SINASC para complementar as variáveis com respostas ignoradas no SINAN. Quando houve discordâncias entre os sistemas, foi priorizado o SINASC.

Analisaram-se variáveis sociodemográficas maternas – faixa etária, raça/cor e escolaridade; e variáveis relativas ao pré-natal/parto – realização do pré-natal (sim/não), momento do diagnóstico de sífilis, esquema de tratamento, testes diagnósticos realizados (teste não treponêmico no parto/curetagem, teste confirmatório treponêmico no parto/curetagem) e tratamento do parceiro. As variáveis idade e escolaridade maternas, raça/cor e número de consultas de pré-natal foram obtidas do SINASC.

O relacionamento entre o SINAN e o SINASC foi realizado pelo programa *OpenReclink* (18), utilizando chaves de blocagem a partir das variáveis “nome da mãe” e “sexo do bebê” e inserindo, nos campos de comparação, o nome da mãe e a data de nascimento do bebê. Para verificar a veracidade de cada par encontrado, analisaram-se as variáveis bairro de moradia, endereço e idade da mãe, data de nascimento e data da notificação. Foram considerados não pareados os casos em que houve dúvida em pelo menos duas variáveis na identificação da gestante, seja por diferença de nome, idade ou endereço.

A incidência da SC foi calculada pela divisão do número de casos novos por ano pelo número de nascidos vivos no mesmo ano, sendo esse resultado multiplicado por 1 000. O número de nascidos vivos foi obtido do SINASC. A análise estatística descritiva foi realizada para o perfil dos casos incidentes de SC quanto às características sociodemográficas, clínicas e de pré-natal. Estimaram-se medidas de frequência para as variáveis categorizadas e medida de centralidade (mediana) para as variáveis quantitativas utilizando o programa *Statistical Package for the Social Sciences* (SPSS) versão 20.0.

A análise da tendência temporal foi feita por meio do programa *Joinpoint Regression,* que permite ajustar uma série de linhas de tendência e seus pontos de junção em uma escala logarítmica, testando as tendências anuais. Foram estimados e testados os percentuais anuais de mudança (*annual percent change*, APC) e seus respectivos intervalos de confiança de 95% (IC95%). Para avaliar a significância estatística, usou-se o teste de permutação de Monte Carlo, que ajusta a melhor linha para cada segmento. Quando não houve mudança, o período foi analisado de forma integral. Para minimizar o efeito de possíveis autocorrelações, utilizou-se a opção disponível no *software* que permite ajustar um modelo de erros correlacionados com base nos dados (*fit an autocorrelated errors model based on the data*) (19).

A estimativa da tendência e da variação anual por transformação logarítmica é defendida para reduzir a heterogeneidade da variância na análise de regressão (15). Para essa análise, a categoria “ignorada” também foi usada em algumas variáveis, a fim de avaliar se a sua evolução temporal seria indicadora de algum padrão.

O projeto da pesquisa foi aprovado pelo Comitê de Ética em Pesquisa da Universidade Federal Fluminense (UFF) (parecer 2601802). Durante a realização do estudo, garantiram-se o anonimato e o sigilo dos dados empregados; os princípios de confidencialidade foram respeitados por meio de procedimentos adotados ao longo do processo de relacionamento probabilístico dos dados. As bases identificadas somente incluíam os campos de identificação necessários para o pareamento. As análises foram realizadas nas bases resultantes não identificadas e os resultados foram apresentados de forma agregada. Os bancos de dados do SINAN e do SINASC foram fornecidos, respectivamente, pela Coordenação de Vigilância em Saúde de Niterói (COVIG) e pela Assessoria de Dados Vitais da Secretaria de Estado de Saúde do Rio de Janeiro para utilização no estudo.

## RESULTADOS

De 2007 a 2016, 754 casos de SC foram notificados e confirmados no município de Niterói, sendo 522 pares identificados nas bases do SINAN e do SINASC. Os 232 casos de SC não pareados por meio do relacionamento foram mantidos na casuística, sendo 51 (21,9%) classificados como abortamento, 38 (16,4%) como natimortos, 23 (9,9%) como óbitos e 120 (51,7%) como nascidos vivos.

Após o relacionamento das bases de dados, houve diminuição da incompletude das variáveis sociodemográficas, mas algumas se mantiveram com percentual ignorado para mais de 5% da amostra. A variável escolaridade materna teve a porcentagem de ignorados reduzida de 49,7% para 18,4%, e a variável cor materna de 7,4% para 6,3% após o relacionamento entre os bancos de dados. O não preenchimento também foi elevado para algumas informações exclusivas do SINAN, como esquema de tratamento materno (10,6%) e tratamento do parceiro (28,7%).

### Características das mulheres, do pré-natal e classificação final

A idade materna variou de 13 a 45 anos (mediana de 22 anos). O maior número de casos de SC ocorreu em filhos de mulheres com menos de 35 anos, com baixa escolaridade (< 8 anos de estudo), de raça/cor parda e que realizaram pré-natal (tabela 1).

O pré-natal foi realizado por 80,8% das mulheres; 39,3% compareceram a sete ou mais consultas. Do total de mulheres, 57,6% obtiveram o diagnóstico de sífilis durante o pré-natal e as demais souberam da infecção apenas durante a internação para o parto ou curetagem, ou esta informação ficou ignorada (tabela 2).

**TABELA 1.** Características sociodemográficas de 754 gestantes com desfecho de sífilis congênita, Niterói, Brasil, 2007 a 2016

Variáveis	No.	%	No.	%
	Antes do relacionamento entre os bancos de dados^a^			Depois do relacionamento entre os bancos de dados^a^
Faixa etária (anos)				
10 a 19	NA	NA	239	31,7
20 a 34	NA	NA	461	61,1
35 ou mais	NA	NA	46	6,1
Ignorada	8	1,1	8	1
Cor da mãe				
Branca	NA	NA	138	18,3
Preta	NA	NA	163	21,6
Amarela	NA	NA	1	0,1
Parda	NA	NA	404	53,6
Ignorado	56	7,4	48	6,3
Escolaridade (anos)				
< 8 anos	NA	NA	473	62,7
>8 anos	NA	NA	142	18,8
Ignorada	375	49,7	139	18,4

***Fonte:*** SINAN e SINASC (2007 a 2016).

a Relacionamento entre o SINAN e SINASC para recuperar dados ignorados. NA: Não se aplica.

**TABELA 2.** Características do pré-natal em 754 gestantes com desfecho de sífilis congênita, Niterói, Brasil, 2007 a 2016

Variáveis	No.	%
Realização de pré-natal		
Sim	609	80,8
Não	71	9,4
Ignorado	74	9,8
Número de consultas de pré-natal		
Nenhuma consulta	27	3,6
1 a 3	42	5,6
4 a 6	144	19
7 ou mais	297	39,3
Ignorado	244	32,4
Diagnóstico de sífilis materna		
Durante o pré-natal	434	57,6
No momento do parto/curetagem	289	38,3
Após o parto	9	1,2
Não realizado	1	0,1
Ignorado	21	2,8
Esquema de tratamento materno^a^		
Adequado	7	1,6
Inadequado	298	68,6
Não realizado	83	19,1
Ignorado	46	10,6
Parceiro tratado		
Não	445	59
Sim	92	12,2
Ignorado	217	28,7
Teste não treponêmico no parto/curetagem		
Reagente	705	93,5
Não reagente	29	3,8
Não realizado	7	0,9
Ignorado	13	1,7
Classificação final do caso de SC		
Abortamento	51	6,8
Natimorto	38	5
SC recente	655	86,9
SC tardia	1	0,1
Ignorado	9	1,2

***Fonte:*** SINAN e SINASC (2007 a 2016).

a De acordo com as normas vigentes no período estudado (1).

O esquema de tratamento foi inadequado em 68,6% das mulheres e não realizado em 19,1%, somando 87,7% das mulheres. Além disso, apenas 12,2% dos parceiros foram tratados (tabela 2). Quanto à classificação final, predominou SC recente (86,9%), seguida de abortos (6,8%) e natimortos (5%) (tabela 2).

### Incidência e série temporal

A incidência média da sífilis congênita foi de 11,9 casos/1 000 nascidos vivos, aumentando de forma significativa e constante de 2007 (4,3 casos/1 000 nascidos vivos) até 2016 (23,2 casos/1 000 nascidos vivos), com mudança percentual anual de 16% (IC95%: 9,9 a 22,4). Em relação à faixa etária, ao final do período, as taxas foram mais elevadas entre as mães adolescentes. Houve intenso e significativo aumento nas mulheres jovens (15 a 19 e 20 a 24 anos), além de aumento moderado naquelas com 25 a 34 anos. Apenas a faixa das mais velhas se manteve estável e com a menor taxa de SC (tabela 3). Em relação à escolaridade, todas as faixas indicaram aumento significativo, sendo o maior em mães com baixa escolaridade, embora aquelas para quem essa informação era ignorada tenham atingido a maior taxa em 2016 (tabela 3).

**TABELA 3.** Incidência de sífilis congênita por 1 000 nascidos vivos e variação percentual anual conforme variáveis sociodemográficas e de pré-natal em 754 gestantes, Niterói, Brasil

Variáveis	2007	2016	Período	Mudança percentual anual (IC95%)	Tendência
Faixa etária (anos)					
10 a 19	5,2	53,0	2007 a 2016	25,2^a^ (12,7 a 39,1)	Aumento
20 a 24	6,3	47,9	2007 a 2016	20,6^a^ (15,0 a 26,5)	Aumento
25 a 34	4,4	10,6	2007 a 2016	7,7 ^a^ (4,8 a 10,6)	Aumento
≥35^b^	0,01	8,1	2008^b^ a 2016	-0,0 (-11,1 a 12,4)	Estabilidade
Escolaridade (anos)					
Ignorada	115,4	551,0	2007 a 2016	5.3 (-6,4 a 18,5)	Estabilidade
< 8	9,1	319,6	2007 a 2016	57,1^a^ (38,7 a 77,9)	Aumento
≥8	1,2	13,7	2007 a 2016	29,4^a^ (24,9 a 34,0)	Aumento
Raça/cor					
Ignorada	29,4	77,4	2007 a 2012	- 20,0 (-36.6 a 1.0)	Estabilidade
			2012 a 2016	60,7^a^ (12,9 a 128,8)	Aumento
Branca	1,7	5,4	2007 a 2016	10,8^a^ (5.3 a 16,6)	Aumento
Preta	14,5	51,7	2007 a 2010	58,5 (-13,9 a 191,9)	Estabilidade
			2010 a 2016	-12,0 (-28,6 a 8,5)	Estabilidade
Parda	6,8	35,4	2007 a 2016	16,8^a^ (9,0 a 25,2)	Aumento
Realização de pré-natal					
Ignorada	70,0	101,7	2007 a 2016	7,8 (-0,4 a 16,6)	Estabilidade
Sim	3,0	20,8	2007 a 2016	17,3^a^ (10,0 a 25,1)	Aumento
Não	14,5	126,4	2007 a 2016	17,6 (-5,6 a 46,4)	Estabilidade

***Fonte:*** SINAN e SINASC (2007 a 2016).

a Significância estatística.

b O ano de 2007 não foi considerado porque ficou como *outlier* na regressão *joinpoint*.

Em relação a raça/cor, as maiores incidências em 2016 foram registradas nas mães com essa informação em branco/ignorada, seguidas das que se autodeclararam pretas (tabela 3). Não houve identificação de tendência significativa de aumento no período de 2007 a 2012 nas categorias de cor preta e ignorada (tabela 3).

Finalmente, em relação à variável da realização de pré-natal, a análise temporal indica que, embora as mães das categorias ignorada e pré-natal não realizado tenham apresentado as incidências mais elevadas, não houve tendência significativa de aumento (*P* = 0,1 em ambas as categorias). De forma inversa, observou-se aumento constante e significativo da taxa de SC na categoria de pré-natal realizado (tabela 3).

## DISCUSSÃO

Este estudo identificou que a incidência de SC foi, além de elevada, crescente em Niterói, município que ocupa o sétimo lugar no *ranking* do IDH no Brasil e que conta com boa cobertura da atenção básica (16, 17). A incidência identificada em 2016 (23,2 casos/1 000 nascidos vivos) foi muito distante da meta da Organização Mundial da Saúde (OMS), adotada pela OPAS para a eliminação da SC (0,5 ou menos casos de SC/1 000 nascidos vivos) (5). O aumento observado em Niterói ao longo do tempo acompanhou as características das regiões metropolitanas do estado do Rio de Janeiro e pode ser explicado, em parte, pelo crescimento da sífilis adquirida entre 2013 e 2017, mais acentuado nas faixas etárias de 10 a 19 anos e de 20 a 29 anos (9). O aumento dos casos de sífilis adquirida também tem sido relatado em outras regiões do Brasil (8) e em outros países (5).

A ocorrência de sífilis e SC em gestantes tem sido associada a fatores sociais, econômicos, de infraestrutura e de acesso aos serviços de saúde, acometendo, muitas vezes, populações de maior vulnerabilidade social (2, 11, 20). Sendo assim, a explicação para os resultados deste estudo pode ser o aumento de iniquidades sociais. As características das mulheres que foram diagnosticadas com SC foram semelhantes às relatadas em outros estudos no território brasileiro, principalmente a baixa escolaridade (2, 13, 21-26) e a cor da pele parda/preta (2, 11, 13, 21-24). Vale ressaltar que a cidade tem um índice de Gini elevado, inferior apenas ao da capital do Rio de Janeiro (16), e uma cobertura da atenção primária elevada, superior a 70% na maior parte do período estudado (17). No entanto, iniquidades sociais podem fazer com que mulheres em situação de vulnerabilidade não sejam captadas e acompanhadas de forma adequada pelos serviços de saúde.

O estudo mostrou que a maioria das mulheres (80,8%) realizou o pré-natal, como também verificado em outros estados do país e no Distrito Federal (4). Entretanto, apenas 39,3% compareceram a sete ou mais consultas; ou seja, o acompanhamento recomendado às gestantes com sífilis não foi alcançado (1). A qualidade do pré-natal das mulheres com sífilis gestacional é baixa em todo o país, tanto pelo início tardio (2, 25) como pelo número de consultas inferior ao recomendado (2, 11, 13, 25) . Um dos reflexos da ausência ou das falhas no pré-natal é o diagnóstico realizado durante a internação para o parto/curetagem ou após o parto em 38,3% das gestantes residentes em Niterói, corroborando os achados de outras localidades (4, 12, 13, 26) . Outro estudo realizado em seis estados brasileiros mostrou que no Amazonas, no Ceará e no Rio de Janeiro a maioria das gestantes (59,7%, 53,3% e 52,1%, respectivamente) foi diagnosticada somente durante a internação para o parto (4). No estado do Rio Grande do Sul, de 2001 a 2012, 0,6% das mulheres não realizou o exame para sífilis no pré-natal, na hora do parto/curetagem ou após o parto (12).

Além do diagnóstico oportuno da sífilis durante a gestação, o tratamento adequado deve ser instituído imediatamente, a fim de evitar a transmissão vertical (1). No entanto, foi identificada uma porcentagem muito baixa (1,6%) de tratamento adequado, resultado ainda inferior ao encontrado em outros estados brasileiros (4, 10, 14), porém semelhante ao encontrado em Palmas (1%), no estado de Tocantins (26). Mundialmente, tanto a incidência da SC como a ocorrência dos desfechos adversos decorrentes da doença estão relacionadas à menor cobertura da atenção pré-natal (7).

Em Niterói, apenas 17% dos parceiros das gestantes cujo desfecho foi de SC receberam tratamento, falha também identificada em outras localidades do país (4, 11, 13, 14, 24, 26) . Uma pesquisa realizada em Porto Alegre mostrou que, mesmo entre os tratados, apenas 12,4% receberam o tratamento adequado. Foi identificada, também, relação entre a realização do tratamento do parceiro e a gestante ter mais de 8 anos de estudo, ter realizado o pré-natal adequado e ter recebido o diagnóstico de sífilis durante o pré-natal (3). É importante salientar que, quando o parceiro não é tratado, pode ocorrer a reinfecção da gestante, sendo imprescindível que os tratamentos ocorram simultaneamente a fim de prevenir a transmissão vertical (1).

Há diversos motivos pelos quais os parceiros não realizam o tratamento. Uma pesquisa em cinco maternidades de Fortaleza, estado do Ceará, revelou que o não tratamento dos parceiros foi explicado em parte por recusa, justificada pelos homens por desconfiança do diagnóstico, por estarem assintomáticos ou por terem medo. Outra parte foi por ocultamento do diagnóstico pela própria gestante, por temer afastamento definitivo do parceiro, para evitar conflitos no relacionamento ou por desconhecimento da importância do tratamento (27). Nesse mesmo estudo, 4,7% das mulheres relataram ter sofrido algum tipo de violência após a revelação do diagnóstico para o parceiro (27).

Mesmo diante da importância do tratamento do parceiro para o controle da SC, essa informação foi excluída como critério de definição de caso de SC em 2017 para fins de vigilância epidemiológica. Anteriormente, a ausência de tratamento era considerada como tratamento inadequado para sífilis materna (28).

Os resultados encontrados neste e em outros estudos mostram que as falhas no pré-natal de mulheres com sífilis estão associadas ao despreparo dos profissionais pré-natalistas, à dificuldade de adesão a protocolos assistenciais, a demoras nos resultados dos exames, à não valorização de títulos baixos de VDRL e à dificuldade na abordagem dos parceiros (29-31). O enfrentamento desse agravo precisa ser feito de forma intensiva e responsável pelos gestores e profissionais da área da saúde.

No Brasil, a taxa de abortamento como consequência da SC varia de 2,2% no Amazonas até 5,6% no Ceará, e o desfecho de natimorto varia de 3,3% no Amazonas, no Distrito Federal e no Rio Grande do Sul, a 10,9% no Ceará (4). Segundo o último boletim epidemiológico, houve menor proporção de abortamento e natimortos no Brasil em 2017 (3,5%; 3,1%) em comparação aos dados de Niterói (6). Assim, encontramos um percentual de natimortos pequeno (5%), semelhante ao achado em Palmas (5,9%), no estado do Tocantins (26). Uma questão importante a salientar é a possibilidade da ocorrência de subnotificação de óbitos por SC.

Em Niterói, a incidência de SC e a velocidade de crescimento da incidência superaram as de um estudo nacional no Brasil, de 2003 a 2008 (13); e de outras localidades brasileiras, como Belo Horizonte, de 2001 a 2008 (23); Rio Grande do Sul, de 2001 a 2012 (12); Mato Grosso, de 2001 a 2011 (14); e outras cinco unidades federadas avaliadas de 2007 a 2012 (4). As tendências mais acentuadas se relacionaram a variáveis sociodemográficas já identificadas em outros estudos brasileiros (2, 11, 13, 21-26), como cor da pele parda ou preta e baixa escolaridade. Até o momento, este é o único estudo que avaliou a tendência temporal por métodos de regressão, de acordo com características sociodemográficas e assistenciais, revelando que a SC está aumentando em grupos populacionais mais vulneráveis socialmente. A frequência de SC em Niterói, localizada em uma área urbana consolidada, com boa cobertura de atenção básica e com IDH muito alto, mostra que, mesmo em municípios com condições de vida favoráveis, existem populações mais vulneráveis do ponto de vista social, suscetíveis a agravos como a sífilis.

Pode-se, ainda, questionar a efetividade da atenção básica do município. Outros estudos mostram que a ampliação da Estratégia Saúde da Família não resultou em melhora no controle da SC (13, 32). É provável que os profissionais que realizam consultas de pré-natal na atenção básica não tenham conhecimento suficiente para o manejo adequado da sífilis na gestação (29-31).

As taxas elevadas e crescentes identificadas em mães adolescentes têm sido pouco relatadas no país (25). Porém, o Boletim Epidemiológico da sífilis apontou que, a cada ano, de 2007 a 2016, houve aumento da taxa de detecção de sífilis em gestantes na faixa etária de 15 a 19 anos no Brasil (8). Além disso, esse grupo populacional tem sido associado a menor cobertura do pré-natal e menor solicitação de testes para sífilis na gestação (33).

Outros países das Américas mostraram que é possível reduzir e até eliminar a transmissão vertical da sífilis (34, 35). O Ministério da Saúde recomenda que sejam instituídos comitês de investigação para prevenção da transmissão vertical em locais que apresentem elevado número de casos de SC (8). Além disso, são necessárias estratégias de saúde pública voltadas às populações socialmente vulneráveis, como fácil acesso a informações sobre a sífilis e sua prevenção e sobre a importância do tratamento, bem como capacitação dos profissionais de saúde para o manejo da sífilis gestacional e para o correto preenchimento da ficha de investigação.

Uma das limitações deste estudo foi o expressivo percentual de informações ignoradas, mesmo após o relacionamento dos bancos do SINAN e do SINASC. A SC é um agravo associado à vulnerabilidade social, e pode ter havido negligência no preenchimento das informações de populações mais vulneráveis. Além disso, as variáveis disponíveis nos sistemas utilizados não abrangem toda a complexidade do agravo, principalmente em relação aos aspectos qualitativos, como o motivo do não tratamento do parceiro. Por outro lado, a análise estratificada de séries temporais para cada variável sociodemográfica e do pré-natal foi inovadora e contribuiu para a melhor compreensão desse relevante problema de saúde pública no Brasil.

Em conclusão, as iniquidades sociais se destacaram na ocorrência de SC. Observaram-se taxas muito elevadas em mulheres pretas e o aumento nas pardas, além de uma tendência temporal de aumento em mulheres jovens, principalmente adolescentes.

É necessária a capacitação dos profissionais de saúde para o manejo da sífilis gestacional, principalmente em populações socialmente vulneráveis, e a realização de trabalho educativo com usuários dos serviços de saúde, principalmente os adolescentes. Ademais, a intersetorialidade de políticas públicas sobre determinantes sociais é primordial para alcançar a meta de eliminação da SC.

Os serviços de saúde devem estar preparados para a captação precoce da gestante no pré-natal e para realizar atividades educativas sobre a sífilis. Além disso, devem ser garantidos os insumos necessários para o diagnóstico e o tratamento do agravo, bem como asseguradas a não discriminação da população e a confidencialidade dos dados dos pacientes.

### Contribuição dos autores.

ALSH e HK conceberam o estudo. ALSH, HK e SCF contribuíram para o desenho do estudo, a análise e a interpretação dos dados e a redação do artigo. SMSB e LAZ contribuíram na análise e na redação. ACR contribuiu na elaboração do banco de dados. Todos os autores revisaram e aprovaram a versão final.

### Agradecimentos.

Os autores agradecem às instituições e aos profissionais que colaboraram para a realização deste estudo. Em especial, agradecem à Coordenação de Vigilância em Saúde de Niterói (COVIG) e à Secretaria de Estado da Saúde pelo fornecimento dos bancos de dados.

### Declaração.

As opiniões expressas no manuscrito são de responsabilidade exclusiva dos autores e não refletem necessariamente a opinião ou política da *RPSP/PAJPH* ou da Organização Pan-Americana da Saúde (OPAS).
